# Healthy Lifestyle and Social Network Prolong Disability-Free Survival in Older Adults With Diabetes

**DOI:** 10.1093/geroni/igab046.702

**Published:** 2021-12-17

**Authors:** Ying Shang, Wei Wu, Abigail Dove, Jie Guo, Anna-Karin Welmer, Debora Rizzuto, Laura Fratiglioni, Weili Xu

**Affiliations:** 1 Karolinska Institutet, Solna, Stockholms Lan, Sweden; 2 Hubei University of Chinese Medicine, Hubei University of Chinese Medicine, Hubei, China (People's Republic); 3 Karolinska Institutet 17175, Stockholms Lan, Sweden

## Abstract

Aim: We aimed to estimate the extent to which diabetes shortens disability-free survival, and identify which factors may prolong disability-free survival in older adults with diabetes. Methods: A total of 2,216 disability-free participants aged ≥60 were followed up to 15 years. Diabetes was ascertained through antidiabetic drug use, medical records, or HbA1c ≥ 6.5%. Disability-free survival was defined as the survival until the occurrence of disability. Data on behaviours (healthy vs. unhealthy), leisure activities (active vs. inactive), and social network (moderate-to-rich vs. poor) were collected at baseline. A favourable (vs. unfavourable) lifestyle profile was defined as the presence of at least one of healthy behaviours, active engagement in leisure activities, and/or moderate-to-rich social network. Data were analysed with Cox regression and Laplace regression. Results: During the follow-up, 1,345 (60.7%) participants developed disability/death. Diabetes was related to the outcome (HR 1.29, 95% CI 1.06–1.57), and shortened 2.15 (1.02–3.27) years of median disability-free survival. Additionally, disability-free survival (95% CI) was shortened by 3.29 (1.21–5.36), 3.92 (2.08–5.76) and 1.66 (0.06–3.28) years for participants with diabetes plus unhealthy behaviours, inactive leisure activities, or poor social network, respectively (reference: no diabetes plus healthy behaviours, leisure activities, or moderate-to-rich social network). Among participants with diabetes, a favourable profile led to a non-significant HR of 1.19 (0.93–1.56) for disability/death and prolonged disability-free survival by 3.26 (2.33–4.18) years than those with unfavourable profile. Conclusions: Healthy lifestyle and/or moderate-to-rich social network attenuates the risk of diabetes on disability/death and prolongs disability-free survival in people with diabetes by 3 years.

